# Microglial Priming and Alzheimer’s Disease: A Possible Role for (Early) Immune Challenges and Epigenetics?

**DOI:** 10.3389/fnhum.2016.00398

**Published:** 2016-08-09

**Authors:** Lianne Hoeijmakers, Yvonne Heinen, Anne-Marie van Dam, Paul J. Lucassen, Aniko Korosi

**Affiliations:** ^1^Swammerdam Institute for Life Sciences, Center for Neuroscience, University of AmsterdamAmsterdam, Netherlands; ^2^Department of Anatomy and Neurosciences, Neuroscience Campus Amsterdam, VU University Medical CenterAmsterdam, Netherlands

**Keywords:** Alzheimer’s disease, microglia, immune system, delirium, dementia, priming, inflammation

## Abstract

Neuroinflammation is thought to contribute to Alzheimer’s disease (AD) pathogenesis that is, to a large extent, mediated by microglia. Given the tight interaction between the immune system and the brain, peripheral immune challenges can profoundly affect brain function. Indeed, both preclinical and clinical studies have indicated that an aberrant inflammatory response can elicit behavioral impairments and cognitive deficits, especially when the brain is in a vulnerable state, e.g., during early development, as a result of aging, or under disease conditions like AD. However, how exactly peripheral immune challenges affect brain function and whether this is mediated by aberrant microglial functioning remains largely elusive. In this review, we hypothesize that: (1) systemic immune challenges occurring during vulnerable periods of life can increase the propensity to induce later cognitive dysfunction and accelerate AD pathology; and (2) that “priming” of microglial cells is instrumental in mediating this vulnerability. We highlight how microglia can be primed by both neonatal infections as well as by aging, two periods of life during which microglial activity is known to be specifically upregulated. Lasting changes in (the ratios of) specific microglial phenotypes can result in an exaggerated pro-inflammatory cytokine response to subsequent inflammatory challenges. While the resulting changes in brain function are initially transient, a continued and/or excess release of such pro-inflammatory cytokines can activate various downstream cellular cascades known to be relevant for AD. Finally, we discuss microglial priming and the aberrant microglial response as potential target for treatment strategies for AD.

## Introduction

Alzheimer’s disease (AD) is characterized by a marked and progressive deterioration of many brain regions, among others those involved in cognitive function and memory. Neuronal loss, synaptic degeneration, accumulation of extracellular amyloid-beta deposits and intracellular neurofibrillary tangles, and an increase in neuro-inflammatory markers are commonly seen in the AD brain (Querfurth and LaFerla, 2010; Rubio-Perez and Morillas-Ruiz, 2012).

Aside from astrogliosis and increased cytokine levels, it is widely accepted that microglia-mediated neuroinflammation contributes strongly to the etiology of neurodegeneration and AD (Yoshiyama et al., 2007; Hickman et al., 2008; Streit et al., 2009; Cunningham, 2013; Mhatre et al., 2015). Indeed, excessive microglial activation and a chronic pro-inflammatory neurotoxic environment contribute to neurodegeneration and cognitive dysfunction (Qin et al., 2007; Bodea et al., 2014; Lim et al., 2015; Wang et al., 2016), similar to what is seen in AD. The hypothesis that neuroinflammation contributes to AD is further supported by studies showing that chronic use of nonsteroidal anti-inflammatory drugs (NSAIDs) appears to delay the onset of AD (Etminan et al., 2003; Vlad et al., 2008).

Although the brain was long considered an “immunologically privileged” site, there is a tight communication between both the peripheral and the central innate immune system, that permits peripheral immune challenges to influence brain function. Common routes of communication between these systems include e.g., a direct access of peripheral cytokines into the brain at sites where the blood-brain barrier (BBB) is leaky. Transport of peripheral signals across the BBB occurs by tightly controlled carrier systems, or via neural afferent pathways such as the vagal nerve. Also, in response to peripheral stimuli, inflammatory factors can be secreted by the BBB itself (Bluthé et al., 1994, 2000; Quan and Banks, 2007; Banks, 2015).

Indeed, evidence for this communication between the peripheral and central innate immune system comes from the classic induction of “non-specific symptoms of sickness” during the course of a systemic infection that makes an individual change his/her appetite, feel like sleeping and retract from his/her daily activities and social interactions (Dantzer and Kelley, 2007). Given the transient nature of most systemic infections, they are generally considered to have limited lasting consequences for brain function in general, or for microglial activation in particular.

Evidence in the last decade, however, indicates that even mild systemic infections can already have deleterious consequences for the brain, particularly when they occur during vulnerable periods, for example during brain development (Adams-Chapman and Stoll, 2006; Bilbo and Schwarz, 2009) or in the aged brain (Dilger and Johnson, 2008; Sparkman and Johnson, 2008).

So far, it remains unclear how a normal homeostatic response to peripheral immune stimuli, may derail into an altered deleterious inflammatory response involving microglia in the brain. Since the occurrence of systemic infections cannot be prevented, it is however crucial to understand the mechanism(s) by which systemic insults contribute to an increased susceptibility to later cognitive and neurodegenerative diseases.

In this review, we will focus on the growing body of evidence from both preclinical and clinical studies suggesting that acute systemic infections may serve as an etiological and predisposing factor for AD. We hypothesize: (1) that systemic immune challenges occurring during periods when the brain is in a vulnerable state, may increase the propensity to induce cognitive decline and AD pathology; and (2) that long-term alterations in microglial function and responsivity, defined as microglial “priming”, may underlie this vulnerability.

We propose that, following infection neonatally or during aging, microglial cells can become persistently more susceptible and responsive to peripheral immune challenges whereas this does not seem to occur during the period of adulthood when microglia appear less sensitive. Furthermore, while first peripheral infections trigger microglia to release cytokines, a secondary insult could induce now “primed” microglia to release an excess of pro-inflammatory cytokines. This microglial response can have detrimental downstream consequences for neuronal functioning that eventually may contribute to cognitive and behavioral deficits and/or accelerate AD pathology.

## Microglia: Immune Cells of the CNS

### Sensitivity of Microglial Cells Throughout Life

Microglial cells in the mature brain continuously, and actively, survey their environment in order to detect potentially harmful or pathological stimuli (Nimmerjahn et al., 2005). Microglia are highly dynamic cells regulating the CNS response to antigens and inflammation (Norden and Godbout, 2013), by rapidly responding to pathogens with expression of specific cytokines, e.g., interleukin 1 and 6 (Van Dam et al., 1992, 1995). Besides continued immune surveillance and mediating inflammatory responses within the brain, microglia contribute to neuronal function (Nimmerjahn et al., 2005; Ransohoff and Perry, 2009; Green and Nolan, 2014; O’Connor et al., 2014; Heppner et al., 2015; Hong et al., 2016) and to brain development, and also to neuronal plasticity throughout life (Sierra et al., 2010; Das and Basu, 2011; Paolicelli et al., 2011).

In contrast to healthy adult brains, the developing brain contains activated microglial cells accompanied by cytokine and chemokine expression (Pousset, 1994; Streit, 2001; Bilbo and Schwarz, 2009; Schwarz et al., 2012). For instance, the microglial derived pro-inflammatory cytokines IL-1β and TNFα are expressed at high levels in the developing CNS (Gilmore et al., 2004; Schmitz and Chew, 2008). Also a TNFα knockout model indicates an active involvement in brain development (Golan et al., 2004). Changes in microglial activation or cytokine expression levels throughout this period can furthermore disturb neuronal development, and affect e.g., cell migration, proliferation, differentiation or synaptic maturation (Giulian et al., 1988; Mehler and Kessler, 1997; Ben-Hur et al., 2003; Gilmore et al., 2004; Deverman and Patterson, 2009).

On the other end of the spectrum, also aging is associated with an aberrant (neuro) immunological response that has been suggested to result from an overall deterioration of the immune system (Sierra et al., 2007; Frank et al., 2010), a process referred to as immune senescence (Ron-Harel and Schwartz, 2009). The alterations in immune cells cause a decreased ability to fight infections and result in a heightened susceptibility of elderly individuals to infectious diseases (Castle, 2000; Zanni et al., 2003). Immunosenescence also occurs in the brain and is thought to involve aging of microglia. Microglial aging is generally reflected by an increased pro-inflammatory state that may result from a switch from a resting microglial phenotype in the adult condition, towards a more reactive and activated phenotype that emerges with increasing age (Luo et al., 2010; Harry, 2013). Given their important, powerful and well-regulated roles in the brain, changes in the levels of pro-inflammatory cytokines during development or aging may impact brain function, as will be addressed below.

### The Acute and Lasting Effects of Perinatal Infection on Microglial Cells

To assess the effects of systemic infections on CNS function in animal models, infections are mostly induced by bacteria as *Escherichia coli (E. coli)* or the gram-negative bacterial component lipopolysaccharide (LPS). Both prenatal and postnatal infections enhance microglial activity and result in increased levels of pro-inflammatory cytokines IL-1β, IL-6 and TNFα in the developing brain within hours after LPS administration (Cai et al., 2000; Paintlia et al., 2004; Billiards et al., 2006; Liverman et al., 2006; Dinel et al., 2014) or *E. coli* infection (Bilbo et al., 2005a). Next to perinatal infection, inflammatory insults in the brain during the perinatal period can also occur in response to injuries in the developing brain, such as ischemia or stroke, or as a (cause and) consequence of preterm birth (for reviews see Hagberg et al., 2012, 2015). For instance, hypoxia-ischemia in neonatal rodents induces a pro-inflammatory state including microglial activation, increased pro-inflammatory cytokine expression and chemokine expression in the first hours to day(s) after the insult (Hagberg et al., 1996; Ivacko et al., 1997; Hedtjärn et al., 2004). These findings are further supported by clinical studies; preterm children e.g., have elevated levels of inflammation-related factors (Duggan et al., 2001; O’Shea et al., 2013), while 6 years later, their immune response is still altered (Lin et al., 2010). It has furthermore been hypothesized that inflammation occurring during this vulnerable period of brain development (Schoderboeck et al., 2009) may lastingly alter, or program, microglial function for the remainder of life (Bilbo and Schwarz, 2009; Krstic et al., 2012).

Indeed, several studies have demonstrated such a “priming-like” effect of a primary exposure to early-life infection on microglial cells, resulting in a more exaggerated microglial response following re-exposure to a similar immune stimulation. The microglial cell activity marker complement receptor 3 (CD11b) was for instance elevated in adult rats that were infected as neonates at P4 (Bilbo et al., 2005a). Also, expression of major histocompatibility complex II (MHCII), a marker of reactive microglia, or the pro-inflammatory cytokine response *per se*, was markedly elevated after a peripheral LPS challenge in adult rats that had been infected during early-life (Bilbo et al., 2005a; Bilbo and Schwarz, 2009).

Given the apparent sensitivity of microglia during the period of early-life, the timing of an immunological challenge is very important when considering possible lasting effects. During development, microglia colonize the (rat) brain from E11 onwards and have acquired a fully adult, ramified morphology around postnatal day 15 (P15; Schwarz et al., 2012; Harry, 2013; Nayak et al., 2014; Reemst et al., 2016), although these time-periods vary in a brain-region specific and sex-specific manner (Pousset, 1994; Harry and Kraft, 2012; Schwarz et al., 2012). Infections taking place outside this developmental time-window will generally have a less profound impact on microglial function as was clearly demonstrated in a study where infection with *E. coli* at P30, but not at P4, failed to induce long-term changes in glial activation and cytokine expression (Bilbo et al., 2006). Clearly, microglial cells are vulnerable to peripheral immune challenges in an age-related manner that can lastingly alter the CNS immune response, whereas mature microglial cells seem to be less vulnerable. The next section addresses a similar shift that occurs in the inflammatory profile of microglia as a result of the normal aging process.

### The Effect of Peripheral Infections on Microglial Cells in the Aging Brain

Although our understanding of glial cell priming following neonatal infections is still limited, evidence exists for similar alterations in microglial function resulting from the aging process (Godbout et al., 2005; Chen et al., 2008; Dilger and Johnson, 2008; Mosher and Wyss-Coray, 2014). The previously described microglial senescence is revealed by an overall upregulation in inflammatory factors in the brain of aged rodents (Lee et al., 1999; Godbout et al., 2005; Sierra et al., 2007; Frank et al., 2010). Indeed, aged microglia show a distinct expression profile, which differs from young mice, or in their response to LPS (Holtman et al., 2015). Moreover, many of the genes that are upregulated in an age-dependent manner in microglia are associated with their activational status or profile, and with the CNS innate immune response (Godbout et al., 2005; Cribbs et al., 2012). This notably also occurs in neurodegenerative diseases, like AD (Li et al., 2014).

Increased expression of the microglial activity markers MHCII and CD11b was found in rodent models of healthy aging, indicative of microglial priming (Godbout et al., 2005; Barrientos et al., 2006; Henry et al., 2009; VanGuilder et al., 2011; Norden and Godbout, 2013). An exaggerated activation of microglial cells is indeed commonly seen in the aged brain after cytokine (Deng et al., 2006) or peripheral LPS administration (Henry et al., 2009) and is often accompanied by enhanced levels of pro-inflammatory cytokines such as IL-1β, TNFα and IL-6 relative to levels found in adult animals after the same stimuli (Ye and Johnson, 2001; Godbout et al., 2005; Sierra et al., 2007).

The aged brain further exhibits deficits in its anti-inflammatory mechanisms and responses, including reduced levels of TNFβ, IL-4 and IL-10 following peripheral infection (Ye and Johnson, 2001; Godbout et al., 2005; Sierra et al., 2007; Wynne et al., 2010; Fenn et al., 2012). These findings are indicative of an altered glial sensitivity/responsivity, or microglial priming, that may result from the process of aging *per se*, and is different from the microglial phenotype found in the healthy adult brain. The underlying mechanisms that trigger these age-dependent alterations in microglial function are unknown and remain an important target for future research.

Another important question is when and how the transition occurs from a well-balanced microglial activational state in the adult brain, into a more reactive and pro-inflammatory profile as found in microglia in aged brains. So far, studies that compared the inflammatory response among young, middle-aged and old rodents, suggest that the increased inflammatory profile in response to systemic challenges is not linear function of age, but appears specific to the advanced age group (Deng et al., 2006; VanGuilder et al., 2011). As no specific time-point of transition appears to exist, it is proposed that the exact timing is individual and based on an interaction between both intrinsic and extrinsic factors (Luo et al., 2010), although the specific modulators of this process remain elusive.

Thus, microglial activation during early-life makes these cells highly sensitive to systemic infections in that period and may shift them towards a priming-like state in a lasting manner. A similar shift in microglial phenotype seems to occur during aging. Although the underlying mechanisms are probably different for both periods, the responses are fairly similar and the parallels are striking. Importantly, in the healthy adult brain, microglial cells seem to be relatively protected against the lasting effects of systemic infections. The next section addresses the impact of peripheral infections on cognition and behavior in vulnerable brains.

## Activation of Primed Microglia by Systemic Infections Impairs Cognitive and Behavioral Functioning

### Microglial Cell Priming *per se* does not Lead to Cognitive and Behavioral Dysfunction

The enhanced activation of the immune system in early-life has repeatedly been linked to a decrease in cognitive functioning later in life, as well as in an increased risk to develop brain disorders, such as autism and schizophrenia (Rantakallio et al., 1997; Bilbo and Schwarz, 2009; O’Connor et al., 2014), but possibly also neurodegenerative disease like AD and Parkinson’s disease (Miller and O’Callaghan, 2008). In addition, an (age-related) increase in pro-inflammatory cytokines is often accompanied by deficits in cognition, brain plasticity, psychomotor co-ordination and an increase in risk for neurodegenerative diseases (Jang et al., 2010; Villeda et al., 2011; Norden and Godbout, 2013).

Together, this indicates that altered microglial function can have a direct and profound impact on brain function. However, exceptions exist too and several rodent studies of early-life infection have failed to find lasting effects on cognition in the Morris water maze (MWM), Y-maze and elevated plus maze (Bilbo et al., 2005b; Spencer et al., 2005; Dinel et al., 2014). Similarly, aged rats did not exhibit profound learning problems compared with adult rats in similar tasks (Barrientos et al., 2006; VanGuilder et al., 2011), but see (Lindner, 1997). Moreover, the variation in cognitive performance increases substantially among aged individuals (Drapeau et al., 2003; Bizon et al., 2009), indicating that while aging is a risk factor for cognitive decline, cognitive decline is no universal characteristic of aging (Lindeboom and Weinstein, 2004; VanGuilder et al., 2011).

### Impaired Cognition and Behavior Following Activation of Primed Microglia by Later Peripheral Infections

Exposure to a second immunological “hit” can alter cognitive function and behavior in perinatally infected as well as aged rodents. The combination of early-life infection with LPS at P14 and subsequent (re-)exposure to LPS during adolescence (P30) or adulthood (P90) revealed impairments in spatial memory performance in mice as tested by the Y-maze (Dinel et al., 2014). In addition, rats infected with *E. coli* at P4 displayed impaired memory in a contextual fear conditioning paradigm after receiving LPS in adulthood (Bilbo et al., 2005b). Exposing P4 infected rats to LPS in adulthood further impaired the long-term (48 h), but not the short-term (1 h) memory for contextual fear experiences, indicating that the deficit was in part mediated by the hippocampus (Bilbo et al., 2006). Again, evidence suggests the existence of a vulnerable time-window, as infection at P30 did not result in LPS-induced memory impairments later in life (Bilbo et al., 2006).

Peripheral immune challenges in aged rodents similarly induce cognitive and behavioral impairments. Chen et al. (2008) e.g., found impaired spatial learning after LPS injection in 22-month old mice compared to younger cohorts, as tested in the radial arm water maze. Moreover, LPS in aged mice induced hippocampal-dependent memory deficits in contextual fear conditioning (Burton and Johnson, 2012). Consistent with this, 24 month old rats exhibited worsened hippocampal memory consolidation in the MWM and in a contextual fear conditioning paradigm after *E. coli* infection relative to 3 month old rats (Barrientos et al., 2006, 2009).

These hippocampal-dependent cognitive impairments were accompanied by additional behavioral alterations. Several studies revealed that aged rodents displayed a prolonged and exaggerated sickness response after immune stimulation with either *E. coli* or LPS, which was characterized by longer anorectic behavior, more weight loss and prolonged social withdrawal behavior (Godbout et al., 2005; Huang et al., 2008; Wynne et al., 2010). In addition to a delayed recovery from sickness, aged mice showed prolonged depressive-like behavior in response to LPS administration compared to adult mice (Godbout et al., 2008).

While also aspects of stress may be relevant here, it is noteworthy that not only peripheral infections may serve as a second “hit” for immune cells. A study of Bilbo (2010) revealed a combined effect of early-life infection and aging, where the process of aging itself was considered a second hit. Rats infected with *E. coli* at P4 were tested for learning and memory at either 2 months or 16 months of age. Neonatal-infected rats exhibited memory deficits in the MWM and a fear conditioning task when tested as aging animals (16 months), suggesting that early-life infection may set off a less successful aging trajectory. The following section will deal with the role of microglia-released cytokines in these cognitive and behavioral deficits.

### Role of Cytokines in Mediating Acute Cognitive and Behavioral Impairments

Almost all cognitive and behavioral deficits that occur following peripheral immune challenges in a “primed” brain condition, appear to be accompanied by an enlarged and prolonged pro-inflammatory cytokine response (Bilbo et al., 2005a; Godbout et al., 2005; Chen et al., 2008; Barrientos et al., 2009; Henry et al., 2009; Püntener et al., 2012). For instance, the cognitive impairments found in early-life infected rodents that were challenged as adults with LPS, were paralleled by a large increase of both IL-1β and TNFα within the first few hours post-LPS in their hippocampus compared to vehicle treated rodents (Bilbo et al., 2008; Dinel et al., 2014). Similarly, a peripheral injection of LPS caused an exaggerated increase of IL-1β, IL-6 and TNFα in brains of aged mice relative to adult ones (Godbout et al., 2005; Barrientos et al., 2006; Chen et al., 2008; Henry et al., 2009), responses that notable lasted up to 24 h. These findings suggest an important role for these particular cytokines in the induction of behavioral and cognitive alterations.

Expression of the cytokine IL-1β in the brain has been implicated in mediating (lasting) central effects following peripheral immune challenges. Both peripheral administration of IL-1β *per se* (Oitzl et al., 1993; Gibertini et al., 1995) or direct injection of IL-1β into the hippocampus (Barrientos et al., 2002) already induce memory deficits, thus linking an enlarged IL-1β response to the hippocampal-dependent impairments seen after immune challenges. In agreement, IL-1 receptors are present throughout the brain including expression in the hippocampal formation (Farrar et al., 1987; Cunningham et al., 1991; Van Dam et al., 1996). Prevention of the IL-1β increase, e.g., by inhibiting IL-1β synthesis (Bilbo et al., 2005a) or by administration of an IL-1 receptor antagonist (Terrando et al., 2010), averted the memory impairments. Furthermore, basal levels of IL-1β of neonatally-infected and aged animals did not differ from their controls prior to a second immune challenge. This agrees with the lack of cognitive and behavioral differences between these groups prior to a secondary stimulus (Bilbo et al., 2005a; Spencer et al., 2005; Barrientos et al., 2009; Dinel et al., 2014).

Next to an exaggerated response in pro-inflammatory cytokines to a secondary immune challenge, also the anti-inflammatory response is altered. Whereas the rise in LPS-inducted TNFβ expression in young animals is normally required to attenuate the IL-1β response, TNFβ mRNA expression remained unaffected after LPS exposure in aged rats (Wynne et al., 2010). Similarly, microglia from aged mice were less sensitive to anti-inflammatory effects of IL-4 (Fenn et al., 2012). In contrast to these data, LPS exposure upregulated anti-inflammatory IL-10 levels in aged mice compared to adults (Henry et al., 2009), although Wynne et al. (2010) found no upregulation of IL-10. As IL-10 is an inhibitor of IL-1β production (de Waal Malefyt et al., 1991; Ledeboer et al., 2002; Lynch et al., 2004), IL-10 might be ineffective as an anti-inflammatory mediator as both appear strongly upregulated in aged animals.

Taken together, activation of primed glial cells by peripheral infections can result in acute hippocampal-dependent cognitive deficits. Whereas sickness behavior usually is an adaptive and reversible process, this response is exaggerated and of an extended duration in animals with primed microglia. The described impairments are mediated by a discordant central inflammatory response, as revealed by enhanced and prolonged production of pro-inflammatory cytokines and a decreased production and effectiveness of anti-inflammatory cytokines. As laboratory animals are housed under (specific) pathogen free conditions, it is important to consider whether these results can be translated to the human situation. Clinical evidence points to similar alterations in cognition in response to systemic infections in vulnerable individuals.

### Systemic Infections Contribute to Cognitive and Behavioral Dysfunction: Evidence from Human Studies

Although infections during early-life can have serious consequences for the developing brain, the underlying mechanisms are not well understood. Similar to animal studies, human studies also suggest that a rise in pro-inflammatory cytokine levels during critical periods can contribute to later-life impairments in cognition (Brown, 2012; Meldrum et al., 2013). Inflammatory factors in preterm infants are found to be associated with cerebral lesions (Duggan et al., 2001) and cognitive impairments in early childhood (O’Shea et al., 2013). Increased levels of IL-1β, TNFα and IL-6 were found in cord blood and cerebrospinal fluid of infants suffering from perinatal complications, including those with sepsis or bacterial meningitis (Miller et al., 1990; Mustafa et al., 1990; Santana et al., 2001). In addition to these acute elevations in cytokine levels, the presence of particularly the pro-inflammatory cytokines in the neonatal blood turned out to be an important predictor of adverse outcomes on neurological functioning in later life (Dammann and Leviton, 2004; Adams-Chapman and Stoll, 2006; Brown and Derkits, 2010; Hagberg et al., 2012), suggesting an important role for early-life microglial activation in mediating long-term adverse outcomes in humans.

Despite these suggestive findings, definitive evidence for a priming-like effect of perinatal infections in humans is lacking. This is due to the fact that human studies are accompanied by technical and ethical difficulties. Several studies further posit the need for a “two-hit" event, building on the hypothesis that a combination of a perinatal infection and a subsequent challenge (e.g., later stress or infection) is required to trigger the manifestation of a disorder, with schizophrenia being one of the best known examples (Feigenson et al., 2014). This suggests that early-life events may (re-)program the brain to become susceptible to challenges later in life (Lahiri and Maloney, 2010). Experimental data to support these hypotheses is, however, limited and evidence for specific reprogramming of the neuroimmune system and microglial cells following perinatal infection in humans is not provided to date.

On the other hand, studies on human aging have provided evidence for an enhanced activation of microglial cells. An activated morphology of microglia has been reported in brains of aged humans (Sheng et al., 1998). Similarly, translocator protein tracers indicate elevated microglial activation in aged as well as AD patients notably in close correlation with cognito-mnemonic scores (Yokokura et al., 2016). Moreover, the gene expression profile in the aged brain indicates a clear upregulation of immune-related genes, including an enhanced expression of IL-1β, TNFα and IL-6 (Cribbs et al., 2012). Since these changes are associated with normal, healthy aging, the question remains whether the immunological response to a subsequent challenge is changed to a priming-like response under aging conditions.

A common phenomenon in elderly is delirium; an acute, transient change in attention and cognition in response to infection or surgery (Wofford et al., 1996; Van Gool et al., 2010). Delirium is strongly associated with an elevated risk of cognitive disorders (Kat et al., 2008; Girard et al., 2010), and the appearance of delirium may further indicate underlying or undiagnosed dementia (Rahkonen et al., 2000; Van den Boogaard et al., 2011). Another study demonstrated increased disturbances in cognitive processing in response to upper respiratory tract infections in older relative to younger individuals (Bucks et al., 2008), indicating an enhanced susceptibility of elderly to cognitive problems after systemic infections compared to adults. The etiology of delirium might further be related to neuroinflammation, as biomarker analysis has associated an elevation of several inflammatory factors (among others, C-reactive protein, procalcitonin and IL-8) with the duration of the delirium or brain dysfunction (McGrane et al., 2011; Van den Boogaard et al., 2011). It has also been suggested that delirium can be the result of microglial overactivation or microglial priming (Van Gool et al., 2010), while it can, according to others, also result from primary astrocytic failure (Sfera et al., 2015). Altogether, these studies indicate that inflammation can play an important role in the manifestation of behavioral deficits in the vulnerable, aged human brain.

Although there is no evidence for consequences of neonatal infection for the later-life neuroimmunological profile in humans, human aging seems to be associated with an increase in the vulnerability to behavioral and cognitive impairments following systemic immune activation. The pathophysiology of delirium and the mechanisms underlying aging-related vulnerability of the brain for inflammatory insults are, however, not clear to date. Although the cognitive and behavioral impairments following immune challenges are generally acute and transient in nature (Murray et al., 2012; Püntener et al., 2012), delirium in elderly also reveals prolonged consequences in the form of cognitive disorders and dementia (Kat et al., 2008; Girard et al., 2010). This raises the question as to whether an inflammatory insult can also have implications for the initiation or progression of neurodegenerative diseases with a suspected neuroinflammatory component. In the next section, we will therefore discuss the possible role of systemic infections in the initiation and progression of AD.

## Systemic Immune Challenges Contribute to Initiation and Progression of AD

Currently, there is little doubt that the increased inflammatory profile after peripheral infections in adults eventually subsides and that microglia return to their pre-activated phenotype. Generally, this is accompanied by the resolution of the acute cognitive and behavioral changes (Cunningham, 2013). If this response is not properly controlled, a lasting overexpression of specific microglia-mediated cytokine profiles may ensue, which may aggravate (aspects of) AD neuropathology (Mrak and Griffin, 2005; Mhatre et al., 2015). Inflammation related factors actually seem to precede the neuropathological changes in AD (Hoozemans et al., 2006), suggesting their involvement already in the early pathological stages. Also other inflammation-related issues have been re-gaining interest recently and are of considerable relevance for AD etiology (Itzhaki et al., 2016). In the next sections, the possible contribution of the microglial response to infection for the initiation and progression of AD pathology will be discussed.

### Systemic Immune Challenges Contribute to Initiation and Progression of AD-Like Neuropathology

AD neuropathology is generally modeled using transgenic models of human genes linked to the development of the familial form of AD, including genes that encode for proteins that accumulate in neurofibrillary tangles and Aβ plaques. Several studies have attempted to examine the effects of peripheral administration of infectious agents on the progression of AD-like pathology in both transgenic animals and wild-type animals. For instance, a single viral administration of mouse hepatitis virus in the triple transgenic mouse model of AD (3x TgAD) resulted in a marked exacerbation of tau pathological features compared to saline injected mice (Sy et al., 2011). Also, a single LPS challenge already altered amyloid precursor protein (APP) expression, parallel to elevated IL-1β and IL-6 levels (Brugg et al., 1995).

Studies on early-life infection have found remarkable evidence for changes in AD-related neuropathological changes already after a single early-life infection. Wild-type mice exposed to viral polyinosinic:polycytidylic acid during late gestation were predisposed to develop AD-like pathology during the course of aging. Moreover, a second systemic immune challenge in adulthood exacerbated this phenotype and drove Alzheimer-like neuropathology, as was revealed by increased APP deposition, altered tau phosphorylation, enhanced glia activation and a chronic elevation of pro-inflammatory cytokines (Krstic et al., 2012). These findings stress the crucial role that inflammation and possibly microglial cell priming effects, e.g., due to maternal or neonatal immune activation, may play in mediating these and other later developing neurodegenerative effects (Krstic and Knuesel, 2013; Knuesel et al., 2014). Similarly, systemic infection with polyinosinic:polycytidylic acid in an animal model of prion disease led to an accelerated progression of the neurodegenerative phenotype (Field et al., 2010), consistent with observations showing that single systemic challenges is a shared environmental risk factor across several brain disorders (Knuesel et al., 2014) and indicating they may be an important factor in the initiation or progression of AD-like pathology.

In addition to these studies of single inflammatory episodes, others using repeated administration of infectious agents also reported effects on the progression of AD-related neuropathology. For instance, repeated peripheral administration of LPS (twice weekly for 6 weeks) to middle-aged 3x TgAD mice led to an increased severity of tau phosphorylation (Sy et al., 2011), in accordance with a similar study performed in younger 3x TgAD mice (Kitazawa et al., 2005). Interestingly, these results were at least partly mediated by a sustained overexpression of the cytokine IL-1β (Ghosh et al., 2013), and the Aβ burden in these same animals was reduced after IL-1β overexpression. Repeated LPS administration for several weeks further affected amyloid processing directly by augmenting amyloidogenic protein expression and APP processing, leading to an increase in Aβ generation compared to saline injected mice (Sheng et al., 2003; Lee et al., 2008). This heightened accumulation of Aβ containing plaque deposition resulted from increased activity of β and γ secretases in response to LPS-induced neuroinflammation (Lee et al., 2008). Lastly, in the 3x TgAD model, twice-weekly LPS administration for 4 weeks increased APP protein accumulation (McAlpine et al., 2009). These findings were overall associated with microglial-induced overexpression of pro-inflammatory cytokines, among which IL-1β and TNFα (McAlpine et al., 2009; Kyrkanides et al., 2011), and could also be blocked by use of anti-inflammatory drugs (Lee et al., 2008; McAlpine et al., 2009). The aggravation of AD related neuropathology following infection can be the direct result of changed microglial cell function, but can also be a consequence of indirect effects of the inflammatory factors on downstream cellular cascades linked to AD processing (for a review see Cunningham, 2013).

Most aforementioned studies make use of repeated LPS administration to mimic peripheral inflammation and an important point is what this stimulus then mimicks. The dosing regimen used in these studies is rather prolonged, making it crucial to investigate whether this mimics a chronic form of systemic inflammation or rather multiple separate infections. Even a milder immunological challenge appears to support the hypothesis that systemic infections contribute to both progression of AD-like tauopathy and Aβ accumulation. Moreover, this relationship seems to be mediated, at least partly, by a microglia-induced exaggerated cytokine response. In this respect, attention has recently been re-drawn to older studies in which specific microbes have been causally implicated in AD etiology (Itzhaki et al., 2016). These included herpes simplex virus, chlamydia etc., that can remain latent in the body for years, can trigger immune responses and are found in human brain as well.

Given the impact that systemic insults may have on AD-like neuropathology, an important point is whether transgenic animal models mimicking a genetic, familial variant of AD, are a good representation of the development of sporadic AD in humans (Kitazawa et al., 2012). Regardless of whether AD is induced sporadically or genetically, all AD cases seem to develop similar neuropathological characteristics, suggesting that animal models are still an important tool to investigate the mechanisms underlying these features (Kitazawa et al., 2012). Peripheral infection in transgenic models, and also in wild-type animals revealed similar effects on AD-related genes and processes, indicating an association between peripheral infections and progression of AD-like pathology.

### Systemic Immune Challenges Exacerbate Cognitive Decline in AD Patients

It has been proposed that the stage, or threshold, for AD onset and severity can possibly be set early in life, since early-life infections may increase the risk of developing AD in later life (Bilbo and Schwarz, 2009; Lahiri and Maloney, 2010). However, clinical and pre-clinical support is limited (Borenstein et al., 2006) and a causal relation is hence difficult to study as it is complicated by the long time period between infection and disease onset.

Several studies on infections throughout life and AD suggest a relationship between systemic inflammation and different hallmarks of AD. A single episode of pneumonia, a common cause of illness worldwide, resulted in an accelerated development of dementia in elderly (Shah et al., 2013). Indeed, the incidence of two or more infections of unspecified type over a period of 4 years was also associated with an increased risk of AD (Dunn et al., 2005). Plasma inflammatory proteins were further found to be increased already 5 years prior to the clinical onset of dementia when compared to age-matched controls (Lim et al., 2015), though this can also be attributed to confounding chronic inflammatory diseases as diabetes or atherosclerosis in these patients.

Still, a comparison of the infectious burden, consisting of herpes simplex virus type 1 (HSV-1), *Borrelia burgdorferi*, *Chlamydophila pneumonia*, *Helicobacter pylori* and cytomegalovirus, between healthy elderly and AD patients revealed a heightened infectious burden in AD patients (Bu et al., 2015) that was associated with worse cognitive function and higher levels of inflammatory cytokines, and higher levels of Aβ. An association between AD neuropathology and HSV-1 was also demonstrated in a study on home-dwelling elderly where HSV-1 was more common among those with lower MMSE scores (Strandberg et al., 2004). Moreover, reactivation of HSV-1, as indicated by heightened levels of anti-HSV antibodies, leads to increased accumulation of Aβ in the brain (Féart et al., 2011; Itzhaki et al., 2016).

Similarly, periodontitis is known to augment levels of circulating TNFα in AD patients (Kamer et al., 2009), although IL-1β and IL-6 were not elevated. Additional inflammatory events in AD patients were associated with elevated baseline levels of TNFα (Holmes et al., 2009) and IL-1β (Holmes et al., 2003) and with an increased rate of cognitive decline over a 2 or 6-month period. On the other hand, individuals with low baseline levels of these cytokines demonstrated more stable cognitive function over time (Holmes et al., 2009). Although such effects were not always found (Holmes and Cotterell, 2009), they support the idea that a heightened inflammatory state exacerbated AD-related cognitive decline.

The above-mentioned studies all suggest a possible role for systemic infections, and subsequently a deregulated microglial response, as an etiological factor relevant for cognitive decline in general, and for AD progression in particular. Treatment of infections and vaccination against common infectious agents may thus, to some extent, protect against the later development or progression of AD (Verreault et al., 2001). However, since AD pathology is known to start many years before the clinical onset of symptoms (Morris, 2005), it is important to consider whether systemic infections contribute to the initiation of AD, or whether they accelerate ongoing processes, which would result in an earlier appearance of clinical signs and symptoms. In the next section, the discordant inflammatory response as a potential target for intervention will be addressed.

## Opportunities to Counteract the Detrimental Effects of Systemic Infections

Given the detrimental effects of systemic infections on brain function and aspects of AD, it is important to think of strategies to counteract these consequences. Considering the above, the microglia-mediated immune response forms an important target. One of the possibilities to interfere with neuroinflammation is by attenuating the aberrant microglial and pro-inflammatory response (Cunningham, 2013; Lim et al., 2015). Indeed, blocking IL-1β significantly reduced neuroinflammation, slowed down cognitive decline and attenuated tau pathology in transgenic mouse models of AD (Kitazawa et al., 2011). Inhibiting TNFα had similar beneficial effects in various animal models (Belarbi et al., 2012; Cunningham, 2013; Camara et al., 2015).

Also, inhibiting other inflammatory cascades, including caspases and prostaglandins was e.g., followed by a decreased neurotoxicity and a reduction of AD-like pathology (Medeiros et al., 2013; Dunn et al., 2015; Heneka et al., 2015). Long-term use of NSAIDs in human, a known inhibitor of prostaglandins, is indicated as a protective factor for AD (Vlad et al., 2008). However, the administration of NSAIDs during the stage of established disease failed to further slow down progression, demonstrating a crucial role for timing of the intervention, given that AD pathology starts several years before clinical symptoms emerge (Morris, 2005; Rubio-Perez and Morillas-Ruiz, 2012).

In contrast to these findings, boosting the pro-inflammatory response could prevent the initiation or progression of established AD neuropathology (Guillot-Sestier et al., 2015b). For instance, chronic over-expression of IL-1β (Shaftel et al., 2007; Matousek et al., 2012), or a reduced amount of anti-inflammatory IL-10 can reduce Aβ plaques in animal models of AD (Guillot-Sestier et al., 2015a). This effect was mediated by an increased ability of microglial cells to phagocyte Aβ plaques and supported by the finding that overexpression of anti-inflammatory IL-10 increased Aβ plaque formation (Chakrabarty et al., 2015).

It is important to understand how an enhanced inflammatory response can exert both adverse and protective effects. The time window of intervention could be one possible explanation, taking into account the pathological stage, age of the animal and basal level of inflammation. Beneficial effects of a boosted inflammatory system is further often investigated using chronic models, i.e., transgenic lines, viral expression regulation, and in relatively young (up to 12 months old) adult animals (Shaftel et al., 2007; Matousek et al., 2012; Guillot-Sestier et al., 2015a). Whether these beneficial effects appear also under acute pathological conditions or in aged individuals with established microglial dysfunction remains to be investigated.

The existence of both beneficial and detrimental consequences of microglia-mediated inflammation for the development AD pathology indicates the complexity of the inflammatory system. Even though enhanced inflammation can have protective effects in animals, systemic infections in both animals and humans generally seem to do more harm than good. The possible transition from healthy microglial activation towards detrimental microglial activation requires a better understanding of the complex machinery by which microglia switch their phenotype. Interestingly, while this may to some extent depend on different microglia subtypes that may differ between different brain regions (Doorn et al., 2015), mechanisms underlying a switch in microglia phenotype and function are currently under investigation, and epigenetic changes have recently been implicated in the changes in microglia-mediated immune responses (Netea et al., 2011, 2016; Garden, 2013).

A study of Cao et al. (2015) revealed that microglia exposed to early-life inflammation carry an “innate immune memory” that is regulated by epigenetic processes including histone acetylation and miRNA signaling. These modifications are thought to allow further activation of microglial cells by subsequent immune challenges, thus the primed response to immune insults (Püntener et al., 2012; Cao et al., 2015). Additionally, both LPS-treated microglia (Cho et al., 2015) and microglia of aged animals (Matt and Johnson, 2015) display hypomethylation of the IL-1β gene promoter, which may explain the heightened microglial activation and pro-inflammatory cytokine levels in response to an immune challenge. Besides, it has been suggested that early-life insults may result in epigenetic regulation of promoter regions of genes involved in AD, thereby possibly explaining enlarged vulnerability of AD after subsequent immune challenges (Lahiri and Maloney, 2010; Krstic et al., 2012).

### Mechanisms of Action

The epigenetic machinery is of particular interest as it may be able to at least partly reprogram the innate immune system, and allow to reverse or to prevent the primed microglial responses. Even though specific reprogramming of the primed cells is currently beyond our capabilities, various factors are known to induce epigenetic changes that can program microglial cell functioning. Microglial cell activation can for instance be suppressed by epigenetic modulation with histone deacetylase inhibitors, preventing an inflammatory state (Kannan et al., 2013). In addition, neonatal handling early in life is an intervention that consists of brief, and often repeated, daily separations of the dam and her pups during the early life period, generally from P3-P10. This is known to enhance the extent of maternal care upon the reunion of the dam with her pups, which is associated with beneficial effects in later life (Meaney et al., 1988; Plotsky and Meaney, 1993; Lesuis et al., 2016). Neonatal handling also increased the expression of the anti-inflammatory cytokine IL-10 in the nucleus accumbens by microglia-specific epigenetic programming (Schwarz et al., 2011). Additionally, neonatal handling of rat pups that had been infected with *E. coli* at P4, prevented both the exaggerated IL-1β response and the memory impairments following exposure to LPS later in life (Bilbo et al., 2007), illustrating the possibility to reverse (aspects of) microglial priming. Although it was not investigated whether this effect was truly established via epigenetic regulation, this might be a likely possibility.

One well-known factor to regulate epigenetic machinery is nutrition (Sezgin and Dincer, 2014). Nutritional intervention may thus be a convenient method for reversing inappropriate expression or silencing of certain genes (Weaver et al., 2005; Lahiri and Maloney, 2010). Methyl group donors (among others the essential dietary components methionine, choline, betaine, vitamin B6 and B12) that are present in the maternal diet during fetal brain development are crucial for modulating offspring microglial function, including programming of the neuroimmune response (Hollingsworth et al., 2008; Canani et al., 2011; Bolton and Bilbo, 2014). Additionally, different nutritional components are currently tested as intervention for different aging-related adversities, including the inflammatory state and cognitive decline (Meydani, 2001; Luchsinger and Mayeux, 2004; Cole et al., 2005; Uribarri et al., 2007; Külzow et al., 2016). Nutritional factors could further reverse the epigenetic alterations that may have resulted from early-life experiences in adulthood (Weaver et al., 2005). However, it is currently unknown whether dietary interventions starting at advanced ages, will exert a similar effect. Particular diets including high levels of omega-3 fatty acids, fruits and vegetables may enhance cognitive functioning and memory accompanied by a reduced risk of developing AD in elderly (Joseph et al., 2009; Jang et al., 2010; Lopez et al., 2011). Besides, an anti-oxidant rich diet slowed down AD progression (Subash et al., 2014). Since many nutrients in these specific diets can be linked to epigenetic processes, it is likely that these nutrients positively affect and/or can possibly normalize an initially “inappropriate” epigenetic status of the older brain (for a review, see Sezgin and Dincer, 2014). However, nutrients may also exert their effects independent of epigenetic modulation, e.g., via (in)direct regulation of microglial activation or cytokine production, or on the general metabolism or stress regulation.

Altogether, these studies indicate that both pharmacological and non-pharmacological interventions may potentially modulate inappropriate microglial cell function by decreasing the (chronic) inflammatory state. Although these results seem promising, it should be kept in mind that microglial function is a complex machinery of interacting mechanisms, whereby intervening in one mechanism may have consequences for the other.

## Conclusion

**Figure 1:**
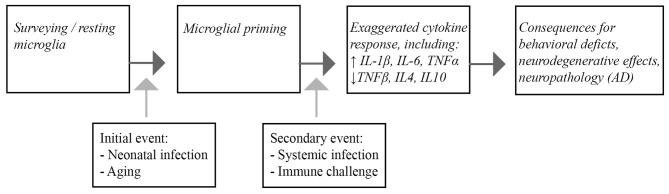
**Schematic representation of the consequences of microglial priming by neonatal infection or aging.** Microglia are generally present as surveying, quiescent cells in the brain. Infection during the neonatal period and aging lead to priming of the microglia. Upon a subsequent inflammatory episode, such as systemic infection or other immunological challenges, the microglial response of the primed cells is exaggerated, leading to increased pro-inflammatory cytokine release. Ultimately the imbalanced inflammatory response ultimatley impacts CNS function that can lead to cognitive dysfunction and neuropathological changes associated with Alzheimer’s disease (AD) in the brain.

In this review, we highlighted parallels between the microglial responses to immune insults in the aging brain, and in studies of early-life infection. In both cases, the normal homeostatic role of microglial cells becomes aberrant and dysregulated, leading to an increased susceptibility of these cells to subsequent immune challenges, an effect known as priming. Interestingly, microglial cells from young adult and middle-aged brains seem to be relatively protected in this respect. Systemic inflammatory episodes, activating the primed glia cells, induce an imbalanced secretion of pro-inflammatory and anti-inflammatory cytokines and have a negative impact on CNS function, as revealed by cognitive deficits and prolonged behavioral alterations (Figure 1).

Moreover, although these impairments are of a transient nature, the exaggerated cytokine expression can also contribute to the induction of neuropathological changes resembling human AD pathology in rodents. Infection-related, exacerbation of pro-inflammatory cytokines is also associated with worsening of clinical symptoms and pathology in AD patients. We therefore may conclude that systemic infections in individuals with primed microglia may be an additional risk factor for AD acceleration. The profound role of microglial cells in mediating the inflammatory profile in the CNS makes them an important target for therapeutic strategies.

A better understanding is needed of how microglial cells respond to their environment and e.g., switch phenotype following (early-life) insults, or during the aging process, and how they interact with the peripheral immune system in response to systemic infections. This is required if we are to target the inflammatory profile in general and microglia in particular. Of course, there are many more risk factors than systemic infections. However, given the aging population, the increasing knowledge about the detrimental effects of neonatal infections, and an environment in which systemic infections are very common, the contribution of systemic immune challenges to the progression of AD can no longer be ignored, and form an attractive target for future research and therapeutic intervention.

## Author Contributions

LH and YH carried out literature search. All authors contributed to the intellectual content and writing of the manuscript.

## Funding

LH and PJL are supported by Alzheimer Nederland, PJL and AK are supported by ISAO and PJL is supported by NWO PRIOMED and the HersenStichting Nederland.

## Conflict of Interest Statement

The authors declare that the research was conducted in the absence of any commercial or financial relationships that could be construed as a potential conflict of interest.
